# Delivery of subcutaneous immunoglobulin by rapid “push” infusion for primary immunodeficiency patients in Manitoba: a retrospective review

**DOI:** 10.1186/s13223-020-00431-3

**Published:** 2020-05-13

**Authors:** Graham Walter, Chrystyna Kalicinsky, Richard Warrington, Marianne Miguel, Jeannette Reyes, Tamar S. Rubin

**Affiliations:** 1 Department of Internal Medicine, University of Manitoba, Winnipeg, Canada; 2 Section of Allergy and Clinical Immunology, University of Manitoba, Winnipeg, Canada; 3 Section of Pediatric Allergy and Clinical Immunology, University of Manitoba, Winnipeg, Canada; 4 Department of Pediatrics and Child Health, University of Manitoba, Winnipeg, Canada

**Keywords:** SCIG, IVIG, Immunodeficiency, Manitoba, Canada, IgG, Retrospective

## Abstract

**Background:**

Both intravenous and subcutaneous human immune globin G (IgG) replacement (IVIG and SCIG, respectively) reduce severe infection and increase serum IgG levels in primary immune deficiency disorder (PIDD) patients who require replacement. SCIG can be administered either with the aid of an infusion pump, or by patients or caregivers themselves, using butterfly needles and a syringe (“SCIG push”). SCIG offers advantages over IVIG, including higher steady state IgG levels, improved patient quality of life indicators, and decreased cost to the healthcare system, and for these reasons, SCIG has been increasingly used in Manitoba starting in 2007. We sought to determine the effectiveness of SCIG push in our local adult PIDD population.

**Methods:**

We conducted a retrospective chart review of all adult patients enrolled in the SCIG push program in Manitoba, Canada from its inception in November 2007 through September 2018. We included patients who were naïve to IgG replacement prior to SCIG, and those who had received IVIG immediately prior. We collected data regarding serum IgG levels, antibiotic prescriptions, hospital admissions, and adverse events during a pre-defined period prior to and following SCIG initiation. Statistical significance was determined via two-tailed t-test.

**Results:**

62 patients met inclusion criteria, of whom 35 were on IVIG prior and 27 were IgG replacement naïve. SCIG push resulted in an increase in serum IgG levels in those naïve to IgG replacement, as well as in those who received IVIG prior. SCIG push also resulted in a statistically significant reduction in number of antibiotic prescriptions filled in the naïve subgroup, and no significant change in antibiotics filled in the IVIG prior group. 8/62 PIDD patients (12.9%) left the SCIG program during our review period for varying reasons, including side-effects.

**Conclusions:**

In a real-life setting, in the Manitoba adult PIDD population, SCIG push is an effective method of preventing severe infections, with most patients preferring to continue this therapy once initiated.

## Background

Primary immunodeficiency diseases (PIDDs) are a heterogeneous group, encompassing inborn errors of both innate and adaptive immunity. An important goal in PIDD treatment is preventing severe infection, which often involves human immune globulin (IgG) replacement. Both IVIG and SCIG have been repeatedly shown to reduce severe infection and increase serum IgG concentration [1–3], and are thus both approved methods of replacement. While the optimal target serum IgG level for patients with PIDD is not known, based on available studies and expert opinion, most clinicians aim to keep serum IgG levels within the normal range for age, with titration upwards to prevent infection on an as-needed basis [4–6]. Some recent studies have shown that IgG levels greater than 7 grams per litre or even higher may be more effective in preventing infection [7, 8]. Although initial FDA recommendations were to use a higher dose SCIG regimen when compared to IVIG to obtain these levels, newer data shows that target serum IgG concentrations can typically be obtained with a 1:1 SCIG:IVIG dosing ratio. This is based on IgG trough data showing that infectious complications are better prevented when SCIG is dosed by this method, rather than the area-under-the-curve pharmacokinetics used by the FDA in their recommendations [9]. This is of clinical and economic importance as it indicates SCIG replacement does not require an excess of biologic product when compared to IVIG. In recent decades, SCIG has been used increasingly, with some data showing increased IgG levels [1–3], improved patient quality of life indicators [2, 10–13], and decreased overall cost to the healthcare system when compared to IVIG [1–5, 10, 12, 14].

Data extrapolated from Atlantic Canada shows that despite the benefits of SCIG, of the 87/100,000 person rate of patients on IgG replacement (2016–2017), 82 received IgG replacement by IVIG, while only 5 received SCIG [13]. This is of local significance, as Canada has the third highest rate of IgG replacement in the developed world at 179 g/1000 persons/year [13], with Manitoba representing the third highest rate per capita of immune globulin replacement within Canada itself.

Because of favourable data regarding SCIG use, as well as the high rate of IgG replacement in Manitoba, SCIG has been increasingly used in this province over the last decade. SCIG can be administered either with the aid of an infusion pump, or by patients or caregivers themselves, using butterfly needles and a syringe (SCIG push) [15, 16]. The push method has been increasingly studied and validated in sites through the United States, Europe, and Canada [17–20]. In fact, many contemporary studies suggest an added benefit of SCIG push over-and-above pump SCIG—including a 2013 retrospective analysis by Shapiro of 173 patients encompassing 1140 hospital visits and approximately 72,000 infusions [18]. This study demonstrated consistently higher serum IgG levels and lower dosing times with push administration, as was previously described [15–20]. A multi-centre randomized controlled trial by Gardulf et al. in 2006 demonstrated that of 60 PIDD patients followed longitudinally on SCIG push, only 8 (13.3%) prematurely discontinued therapy - 1 was lost to follow-up due to travel out of the country, 1 suffered a suspected systemic reaction, 2 withdrew consent for unknown reasons, 1 suffered moderate localized reactions, 1 was unable to obtain satisfactory IgG levels, and 2 were removed due to protocol violation (history of anaphylaxis to IVIG and renal failure) [3].

Since 2007, Manitoba PIDD patients requiring IgG replacement have had the option to receive monthly IVIG in hospital, SCIG via an infusion pump, or SCIG via self-infusion. At the program’s inception, SCIG infusions were established with the lower concentration product available at the time (16% IgG *Vivaglobin*) [21]. When *Hizentra* (20% IgG) [22] became commercially available in 2010, all SCIG patients were transitioned to this, and more recently, a similar transition occurred from *Hizentra* to *Cuvitru* with changes to the Canadian Blood Services formulary [23].

Although the effectiveness of SCIG push replacement has been investigated before, we sought to investigate the effectiveness and drop-out rates associated with this form of replacement in our local Canadian population.

## Methods

We conducted a retrospective chart review of all patients enrolled in the SCIG push program in Manitoba from its inception in November 2007 through August 2018.

We included patients ≥ 18 years old during our study period, with a diagnosis of PIDD as their indication for IgG replacement. Patients must have been receiving SCIG push as their exclusive form of replacement for a period of ≥ 12 consecutive months. Patients could be either IgG replacement naïve at the time of starting SCIG push, or previously receiving IVIG immediately prior to starting SCIG. Exclusion criteria included administration of SCIG via infusion pump or receiving IVIG at any point during the review period. In the case of patients who left the SCIG program and subsequently resumed this method of IgG replacement, only their first trial of SCIG was included in our analysis, assuming that they met other inclusion criteria.

We extracted patient demographics such as age, weight, and SCIG dose. Comorbidities such as bronchiectasis and chronic rhinosinusitis were recorded. Specific PIDD diagnoses were documented, using diagnostic criteria from the American Association of Allergy, Asthma, & Immunology (AAAAI) Immunodeficiency Practice Parameter where possible [24]. Individual patient IgG levels were obtained 6 months before and 12 months after starting SCIG. These levels represent steady-state concentrations with regard to SCIG, and trough concentrations (pre-infusion collection) with regard to IVIG. Laboratory results were obtained from electronic medical records and from hospital paper charts.

We used data from Manitoba’s Drug Programs Information Network (DPIN) to compare antibiotic prescription courses filled by each patient in the 12 months prior to and 12 months after starting SCIG. Frequency of individual patient hospitalization was obtained from review of patient clinic letters and electronic medical records. We reviewed patient-reported adverse events and reasons for discontinuation from the program, where relevant. The data were summarized by group (IVIG prior vs. IVIG naïve) and period (before and after conversion from IVIG to SCIG, where applicable) using conventional descriptive statistics (counts and percentages, means and standard deviations). The groups were compared using t-tests on the paired differences, considering p-values less than 0.05 to be indicative of statistically significant differences associated with the group effect on these changes. Missing values (typically due to data prior to 2007 which was not available for analysis) were not included, and no attempt was made to account for multiple testing.

## Results

**Fig. 1:**
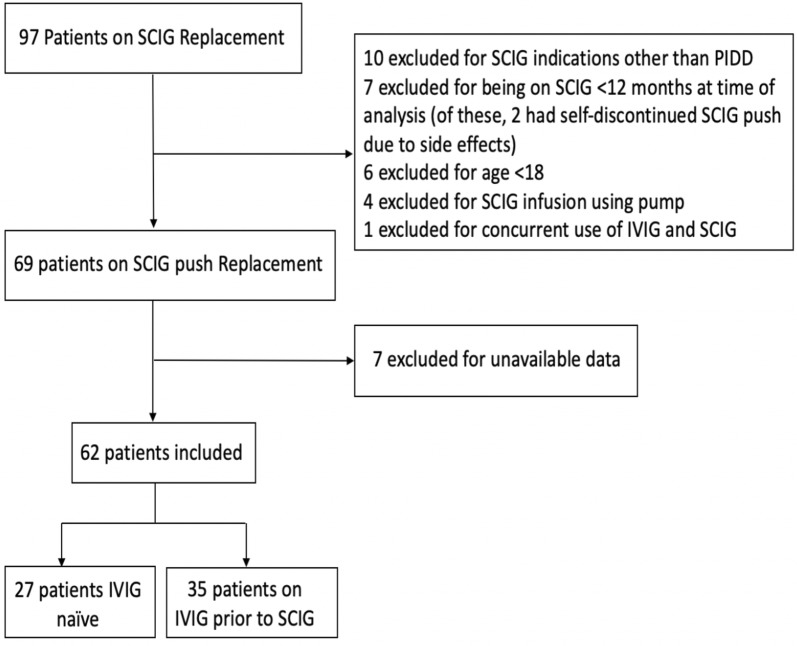
Inclusion and exclusion criteria

62 patients were included [27 IgG replacement naïve, 35 on IVIG in the period preceding SCIG (Fig. 1)]. Common variable immune deficiency (CVID) (38.7%) and IgG subclass deficiency (IGGSD) (33.9%) were the most frequently represented diagnoses across both groups. Within the IgG naïve group, 55.6% of patients had bronchiectasis, chronic rhinosinusitis (CRS), or both, while within in the IVIG-prior group the proportion was 45.7% (Table 1).

**Table 1:** Background characteristics of patients treated with SCIG push

	IVIG naïve (N = 27)	IVIG prior (N = 35)
Age at SCIG initiation, mean (range)	50 (30–78)	51 (26–80)
SCIG dose [g/kg/week] (range)	0.14 (0.06–0.29)	0.15 (0.09–0.22)
Sex		
Female	17 (63%)	23 (66%)
Diagnosis		
CVID	13 (48%)	11 (31%)
IgGSD	8 (30%)	13 (37%)
Hypogammaglobulinemia NOS	5 (19%)	7 (20%)
SAD	–	1 (3%)
X-linked agammaglobulinemia	–	1 (3%)
Unspecified humoral immunodeficiency	1 (4%)	2 (6%)
Comorbidities		
Bronchiectasis, CRS, or both	15 (56%)	16 (46%)

*IGGSD* IgG subclass deficiency, *CVID* common variable immunodeficiency, *SAD* specific antibody deficiency, *NOS* Not otherwise specified, *CRS* chronic rhinosinusitis, *g*  grams, *kg* kilograms

**Fig. 2:**
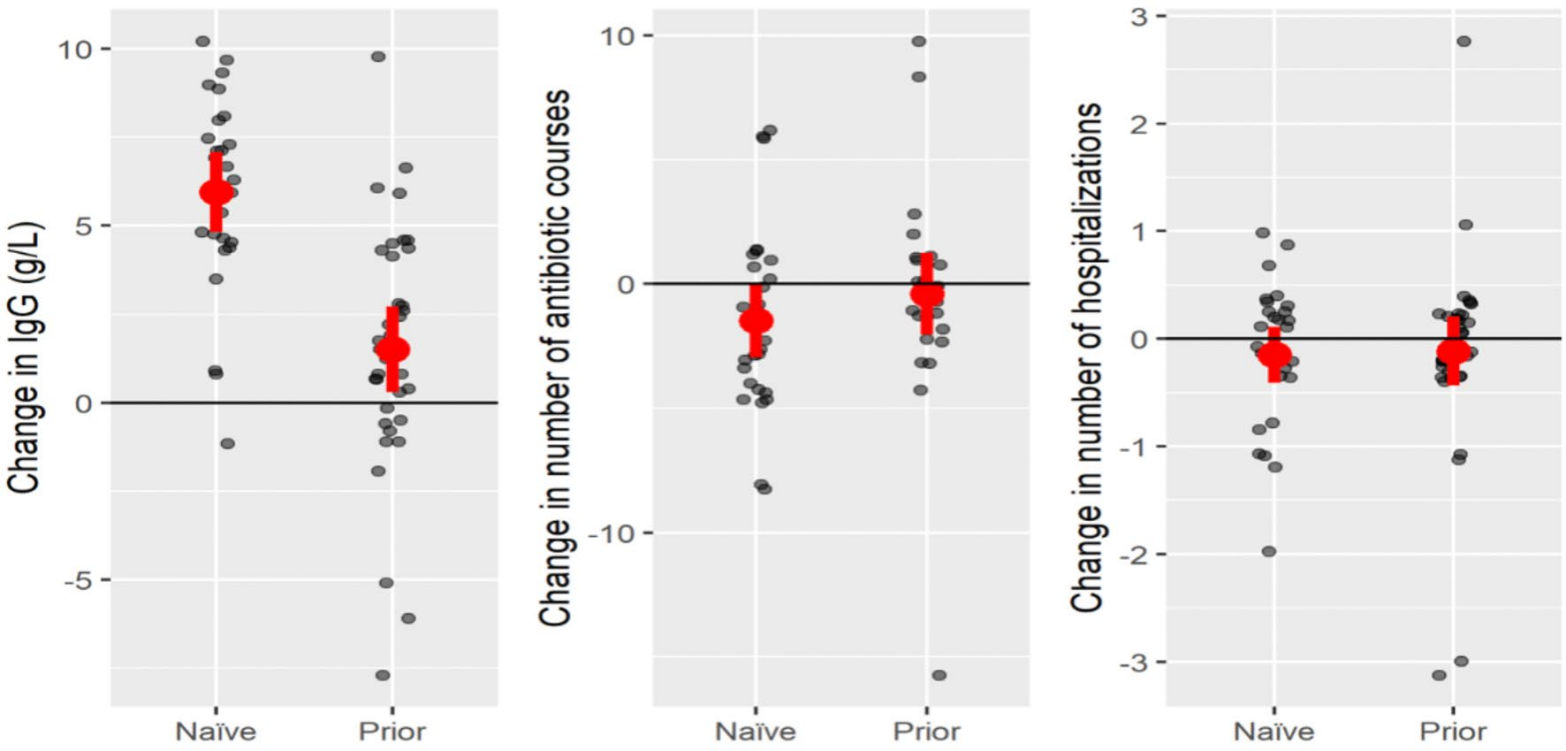
SCIG push replacement in IVIG naïve patients, and those prior on IVIG

IgG administration via SCIG push provided an adequate steady-state IgG, and resulted in a statistically significant reduction in antibiotic prescriptions filled in the IgG replacement naïve population (Table 2, Fig. 2). With regard to Manitoba adult PIDD patients who were previously on IVIG replacement, SCIG push also provided a statistically significant higher steady-state IgG concentration, and resulted in a similar number of hospitalizations, and antibiotic prescriptions filled. The lack of increased antibiotic prescriptions and hospitalizations in the SCIG push population suggests that this is as effective as IVIG in our local population (Table 3, Fig. 2).

**Table 2:** SCIG push replacement in IVIG naïve patients

	IVIG naïve			
	Average value prior* (range)	Average value 12 months post SCIG (range)	Mean difference (standard deviation)	p-value
Serum IgG level (g/L; normal range 6.9–16.2)	4.87 (< 0.33–12.30)	10.83 (5.85–16.1)	+ 5.96 (2.82)	*< 0.0001*
Antibiotic prescriptions filled	5.67 (1–14)	4.19 (0–16)	− 1.48 (3.70)	*0.048*
Number of hospitalizations	0.37 (0–2)	0.22 (0–1)	− 0.148 (0.662)	0.256

*6 months prior with respect to IgG trough serum levels and 12 months prior with respect to antibiotic prescriptions and hospitalizations Values in italics indicate statistical significance as defined in methods

**Table 3:** SCIG push replacement in patients on prior IVIG

	IVIG prior			
	Average value prior* (range)	Average value 12 months post SCIG (range)	Mean difference (standard deviation)	p-value
Serum IgG level (g/L; normal range 6.9–16.2)	10.72 (6.76–16.80)	12.22 (4.99–16.20)	+ 1.50 (3.54)	*0.017*
Antibiotic prescriptions filled	3.93 (0–20)	3.54 (0–16)	− 0.393 (4.24)	0.628
Number of hospitalizations	0.31 (0–4)	0.20 (0–3)	− 0.114 (0.932)	0.473

* 6 months prior with respect to IgG trough serum levels and 12 months prior with respect to antibiotic prescriptions and hospitalizations. Values in italics indicate statistical significance as defined in methods

During the review period, 8 of the 62 patients studied discontinued SCIG therapy following at least 12-months of SCIG push as their sole form of IgG replacement; 4 each from the IVIG prior and IgG naïve subgroups. Of these 8 patients, 7 transitioned back to IVIG, and 1 chose to discontinue IgG replacement entirely. Reasons for SCIG push discontinuation included inconvenience of IgG replacement in general (1 patient), inconvenience of SCIG replacement schedule in particular (1 patient), no reason given though poor adherence was noted (2 patients), infection perceived by the patient to be SCIG-related (1 patient), pregnancy (1 patient), infusion pain related to prior surgical scars at infusion site (1 patient), and fatigue perceived to be related to infusion (2 patients). The patient who disliked the SCIG replacement schedule discontinued IgG replacement altogether. Five patients who had transitioned back to IVIG remained on IVIG therapy at the end of the review period. However, one of these patients was pregnant, and planned to resume SCIG post-partum. Two patients who had initially transitioned back to IVIG eventually chose to resume SCIG.

## Discussion

Our study represents the first local analysis of SCIG push in the Manitoba population, and, as far as we know, is the first analysis of SCIG push effectiveness for primary immunodeficiency in Canada. Our study agreed with prior studies showing that SCIG by push prevented infection in a PIDD population, and was well accepted by patients, with a relatively low rate of attrition [1, 5, 6, 10, 12, 20, 25, 26]. In our study, SCIG push patients had a statistically significant increase in their serum IgG levels whether they had transitioned from IVIG, or had been started on SCIG push as an initial form of Ig replacement. It should be noted, however, that differences in serum IgG concentration within the IVIG prior group may have been confounded by the difference in measurement timing (i.e. steady state concentrations for SCIG and trough concentrations for IVIG). SCIG push patients who were naïve to replacement also had a reduction in antibiotic prescriptions after treatment compared to before treatment, and those who were on IVIG prior had no increase in the rate of antibiotic prescription on SCIG, suggesting that SCIG replacement was no less effective compared to IVIG.

Given that SCIG push is performed at home by either patients or their caregivers, whereas IVIG is scheduled on a monthly basis and administered by nursing staff at hospital, we presumed that adherence might be a significant issue with SCIG replacement. Although we did not attempt to extract adherence data, the adequate IgG steady state values and reductions in antibiotic courses and hospitalizations in both groups suggest that self-administered SCIG push is effective in a real-life setting. The reduction in serum IgG concentration seen by the outliers in Fig. 2 are likely explained by adherence issues. Further, our data demonstrated that patient adherence was high enough to obtain timely IgG steady state levels in the vast majority of patients. Findings from our study have implications for the treatment of PIDD patients in other regions of Canada with similar challenges, including rural or remote populations [13].

The rate of attrition from SCIG therapy in our population was low (8 of 62 patients, representing 87.1% patient adherence), suggesting patient satisfaction with this method of treatment. This is comparable to the Gardulf study, which demonstrated 86.7% patient adherence to SCIG push treatment [3]. Shapiro also demonstrated that 78.3% of pump SCIG patients chose to remain on this method of replacement, and 81.3% of SCIG push patients chose to do the same, with good rates of adherence as demonstrated by satisfactory IgG steady state levels [18]. In our study, 2 patients did not meet inclusion criteria because they discontinued SCIG push therapy prior to completing 12 sequential months of therapy. In their cases, they discontinued therapy due to perceived side-effects, but it is notable that one of those patients eventually chose to resume SCIG replacement. This patient was IVIG naïve at the time of SCIG push replacement initiation, while the latter patient was on IVIG prior and chose to return to this method (Fig. 1).

Although SCIG has been widely adopted throughout Europe since the early 1990s, infusions have been facilitated by pumps rather than by using the push technique, and thus most efficacy and cost-effectiveness data for SCIG is based upon pump-facilitated infusions [27–31]. This may be a reflection of features of the European healthcare system, including the proximity of patients to health care centres. Data on SCIG push to date has primarily come from several analyses by Shapiro et al. in an American PIDD population, and have demonstrated the benefit of push replacement over-and-above pump with respect to serum IgG levels, patient satisfaction, number of infusion sites per patient, and infusion time [16–18].

Unfortunately, despite a growing body of knowledge, there is a paucity of data on SCIG push in the adult Canadian population. A single 2012 study by Martin et al. evaluated the economics of the Vancouver home push infusion program compared to using pumps for local PIDD patients [14]. Although the analysis was in favour of the push technique, it was solely an economic assessment and did not address real-life efficacy of this method of replacement. Ducret et al. also published a similar study demonstrating pharmacoeconomic benefits of SCIG (both push and pump) in a Quebec population, but the patients were exclusively pediatric [32].

Given that Manitoba has a high relative use of IgG replacement [13], both economic and effectiveness data for various replacement techniques is important, and should ideally be investigated in the Canadian and Manitoba-specific context. Manitoba occupies a unique place within Canada’s healthcare landscape, as a single academic institution in the province’s capital (Winnipeg) services almost all immunodeficiency patients from a large catchment area extending through Northwestern Ontario and Nunavut.

Although a number of IVIG infusion clinics exist outside of Winnipeg, this is still a service limited to a minority of centres due to the wide population distribution and low density; in fact, of the 1,278,365 people residing in Manitoba, population density is a mere 2.3 persons/km^2^ compared with Canadian averages of 3.9 persons/km^2^ [33]. Furthermore, a significant proportion of the population serviced by our centre live rurally or in remote locations, with 42.6% of Manitoba residents living rurally in general, and 5.7% living in our Northern Health Region [34]. Our study population follows this trend, with 39/62 patients studied living within Winnipeg itself and 23/62 living elsewhere (rural Manitoba and Western Ontario), travelling an average of 197 km to seek care in Winnipeg. Thus, an alternative to hospital-based therapy such as home-administered Ig replacement is a highly appealing and practical prospect. Furthermore, human and material resources required for the use of mechanical infusion pumps are limited.

In Europe and Japan, a long-term efficacy review of 7 phase 3 trials of *Hizentra* demonstrated that of the 125 unique patients studied, 43 discontinued SCIG, most commonly due to withdrawal of consent (n = 20), adverse events (n = 12), and other reasons including patient non-adherence (n = 11). Unfortunately, similar data involving IVIG adherence in the PIDD population for comparison is scarce. In the 2017 IDEaL patient registry, of 383 patients studied (3758 doses), 6% of SCIG doses were missed, 4% were delayed, and 0.4% were “incomplete”. The corresponding numbers in the IVIG patients were 1%, 4%, and 1%, respectively [35]. This is in keeping with the AAAAI update on the use of immune globulin in human disease, which discourages the use of SCIG in patients who have previously demonstrated non-adherence to treatment [36].

Our conclusions are limited by the fact that the number of antibiotic prescriptions filled does not reflect severity or type of infection, duration of antibiotics, or confirm a true bacterial infection. Furthermore, we used DPIN and EPR records to determine antibiotics filled and hospitalizations, which do not capture hospitalizations at community hospitals within the province, nor does it capture prescriptions filled outside the province or antibiotics administered via the emergency department or as an inpatient. Although there appeared to be a trend toward decreased hospitalization after starting SCIG, there was a low rate of baseline hospitalization detected in our population in general, and the change in rate of hospitalization before and after starting SCIG was not statistically significant.

Another limitation of our study is the fact that, in the years since the Manitoba SCIG program inception—and depending on the commercial SCIG product available at our centre—patients in the program self-infused with either *Vivaglobin* (16% IgG) [21], *Hizentra* (20% IgG) [22], or *Cuvitru* (20% IgG) [23]. Due to the small numbers in the present study, we did not attempt to stratify results based on the products used, although it has been previously shown that the 20% IgG products result in a decrease in number of infusion-sites in a significant proportion of patients without compromising effectiveness, side-effect profile, convenience, or global satisfaction [37].

A further limitation, due to the retrospective nature of this study and the lack of a centralized database of immune deficient patients receiving IVIG, is that we were unable to compare the number of patients initiating, maintaining and discontinuing IVIG with the number of patients initiating, maintaining and discontinuing SCIG over the same time period. In general, there are currently approximately 100 Manitoba patients receiving IVIG through the allergy/immunology clinic, the vast majority of whom receive it for the indication of immunodeficiency. Unfortunately, however, we are unable to ascertain if more patients initiated IVIG or SCIG since SCIG became available in our province. As well, initiation of SCIG over IVIG (even if that was the patient or provider preference) was sometimes limited by product supply or availability of nursing training support for patients. It is certainly also possible that patient characteristics (for example age, underlying immune deficiency diagnosis, proximity to tertiary care centre, etc.) impacted both patient and provider choice for method of replacement. Optimally, a future prospective study would be designed to examine the rate of initiation of SCIG versus IVIG in our primary immune deficiency population, as well as attrition rates for each, and rates of switchover between these methods of replacement.

Finally, a major challenge associated with studying this patient population is the heterogeneity of the immunodeficiency population itself. A genetic diagnosis was not known for the vast majority of patients, and the severity of the immune defect and associated comorbidities within the IVIG naïve and the IVIG prior groups—as well as across the two groups—may have varied.

In our study, there was a high proportion of patients with the diagnosis of IGGSD, which is a controversial diagnosis [38]. We acknowledge that there are ongoing questions regarding consistency of IgG subclass measurement across different laboratories, a lack of age appropriate reference ranges for IgG subclasses, and, in some cases, a lack of correlation between subclass deficiency and documented objective antibody dysfunction. All of these issues have resulted in further scepticism concerning IgG subclass deficiency as a clinical diagnosis and indication for immune globulin replacement [39].

Generally, immune globulin replacement is only considered in IGGSD patients if they demonstrate a “significant antibody deficiency” or “recurrent infections”, based upon 3 studies demonstrating benefit in quality of life and reduction in infection [36, 38, 40–42]. Indeed, a recent study of a somewhat analogous population (patients with specific antibody deficiency), found that while prophylactic antibiotics and immune globulin replacement therapy were equally effective as first line in preventing infections in this population, patients who fail prophylactic antibiotics would benefit from immune globulin replacement therapy [43]. In general, immune globulin replacement for IGGSD patients in our population would only have been recommended by their immunology specialist if they were experiencing severe infection, or after demonstrated failure of antibiotic prophylaxis. In some cases, patients we classified as IGGSD had a history of impaired polysaccharide response. However, if we were unable to confirm this in their medical records objectively, they were classified based on results that were available. A future study formally comparing antibiotic prophylaxis and immune globulin replacement in Manitoba Patients with IGGSD and/or specific antibody deficiency would be valuable, particularly since Canada in general, and Manitoba in particular has a high per capita usage of immune globulin, which is a limited and valuable resource [13].

In addition to variability of underlying diagnosis within our study, pre-existing comorbidities such as bronchiectasis and chronic rhinosinusitis can contribute to an increased need for antibiotic prescriptions regardless of immune globulin replacement, and thus frequency of antibiotic prescription may not actually reflect efficacy of the Ig treatment itself. By comparing antibiotic prescriptions for individual patients before and after starting on SCIG, we aimed to reduce the confounder of bronchiectasis and chronic rhinosinusitis, although these patients may have skewed the averages for the group as a whole.

Going forward, further studies could explore patient-centred outcomes associated with using SCIG push in PIDD patients, including assessing quality of life measures. Although patient satisfaction data exists (quality of life, life quality index, health related quality of life) for European, Japanese, and American patients, this data is lacking for our local population in the setting of our unique healthcare system [10–12, 20, 44–47]. As new formulations of SCIG products become available in Canada—including those that require less frequent administration [48]—effectiveness, attrition data, economics, and quality of life analyses will be required for the Canadian context.

## Conclusions

In a real-life setting, in the Manitoba adult PIDD population, SCIG administration via push is an effective mode of IgG replacement, with most patients preferring to continue this therapy once initiated. In the context of a publicly funded Canadian healthcare system with limited resource allocation, and which services rural and remote locations, SCIG push may be a preferred option compared to IVIG or administration by pump.

## Data Availability

The datasets generated and/or analysed during the current study are not publicly available due to individual health data privacy, but are available from the corresponding author upon reasonable request.

## Abbreviations

AAAAI: American Association of Allergy, Asthma, & Immunology
CRS: Chronic rhinosinusitis
CVID: Common variable immune deficiency
DPIN: Drug Programs Information Network
IgG: Immunoglobulin G
IGGSD: Immunoglobulin G subclass deficiency
IVIG: Intravenous immune globulin
NOS: Not otherwise specified
PIDDs: Primary immunodeficiency diseases
SCIG: Subcutaneous immune globulin
